# Efficacy of primary drainage by endoscopic ultrasound‐guided biliary drainage for unresectable pancreatic adenocarcinoma

**DOI:** 10.1002/jgh3.12732

**Published:** 2022-04-12

**Authors:** Tomohiro Tanikawa, Katsunori Ishii, Ryo Katsumata, Noriyo Urata, Ken Nishino, Mitsuhiko Suehiro, Miwa Kawanaka, Ken Haruma, Hirofumi Kawamoto

**Affiliations:** ^1^ Department of General Internal Medicine 2 Kawasaki Medical School Okayama Japan

**Keywords:** biliary drainage, endoscopic retrograde cholangiopancreatography, endoscopic ultrasonography‐guided biliary drainage, metal stent, obstructive jaundice

## Abstract

**Background and Aim:**

Obstructive jaundice induced by pancreatic adenocarcinoma is typically treated with biliary drainage with endoscopic retrograde cholangiopancreatography (ERCP)‐guided biliary drainage (ERCP‐BD). Recently, endoscopic ultrasonography‐guided biliary drainage (EUS‐BD) was employed as an alternative method after ERCP‐BD failed. We aimed to determine the efficacy and safety of EUS‐BD for primary biliary drainage.

**Methods:**

Between December 2011 and February 2019, at Kawasaki General Medical Center, we retrospectively enrolled 33 patients who had undergone endoscopic biliary drainage with a metal stent, in a first attempt to relieve obstructive jaundice caused by unresectable pancreatic adenocarcinoma. We compared the technical and clinical outcomes between ERCP‐BD and EUS‐BD.

**Results:**

Twenty‐three patients underwent ERCP‐BD and 10 underwent EUS‐BD. Both groups achieved 100% technical success. The clinical success rates were similar between the groups: 91% (21/23 patients) for ERCP‐BD and 100% (10/10 patients) for EUS‐BD (*P* = 0.48). Biliary obstruction recurred in 6/23 patients (26%) treated with ERCP‐BD and 1/10 patients (10%) treated with EUS‐BD (*P* = 0.40). Other adverse events occurred in 4/23 patients (17%) in the ERCP‐BD group and 1/10 patients (10%) in the EUS‐BD group (*P* = 0.99).

**Conclusion:**

We suggest that EUS‐BD could be employed for primary biliary drainage in patients with obstructive jaundice caused by unresectable pancreatic adenocarcinoma.

## Graphical abstract

Endoscopic ultrasound‐guided biliary drainage could serve as the primary biliary drainage option for obstructive jaundice caused by unresectable pancreatic adenocarcinoma. This approach could be particularly favorable in patients with duodenal stenosis or tumor involvement at the orifice of the cystic duct.

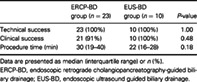

## Introduction

Pancreatic adenocarcinoma is one of the most aggressive cancers, and unresectable pancreatic adenocarcinoma has a particularly poor prognosis. Obstructive jaundice is a typical morbidity induced by pancreatic carcinoma. Obstructive jaundice can aggravate the patient's quality of life; however, it can be improved with biliary drainage. Currently, the first‐choice treatment for this morbidity is an endoscopic stent placement; metal stents are often employed for unresectable cases because of their long‐lived patency. To obtain favorable outcomes, we generally insert a metal stent through the duodenal papilla, in a procedure termed endoscopic retrograde cholangiopancreatography (ERCP)‐guided biliary drainage (ERCP‐BD). However, in difficult cases, duodenal stenosis might prevent even expert hands from approaching the papilla of Vater. To address this problem, in 2001, Giovannini *et al*. introduced an approach called endoscopic ultrasound‐guided biliary drainage (EUS‐BD).^1^

EUS‐BD demands a high level of endoscopic skill. It is performed by expert endoscopists. The technical and clinical success rates of EUS‐BD were reported to be 90.9–100% and 75–100%, respecively.^2^, ^3^, ^4^, ^5^, ^6^, ^7^, ^8^, ^9^, ^10^, ^11^, ^12^, ^13^ Currently, EUS‐BD is employed as alternative approach after ERCP‐BD fails. EUS‐BD was associated with an adverse events rate of 6.3–19%.^2^, ^3^, ^4^, ^6^, ^7^, ^8^, ^9^, ^11^, ^12^, ^13^ EUS‐BD can cause bleeding, bile peritonitis, stent dysfunction, and stent migration, which can lead to severe morbidity and mortality. These adverse events sometimes require a surgical intervention for rescue. Despite these drawbacks, EUS‐BD is a useful drainage procedure.

Another alternative is percutaneous transhepatic biliary drainage (PTBD). This procedure is also employed after ERCP‐BD failure. However, PTBD requires external catheterization, which can impair the patient's quality of life. Furthermore, Iwashita *et al*. reported that EUS‐BD was comparable to PTBD in terms of efficacy and safety for distal biliary obstruction due to a malignant tumor.^14^ Considering these facts, we prefer the EUS‐BD approach to the PTBD approach, after ERCP‐BD failure. The aim of this study was to retrospectively evaluate the efficacy and safety of primary biliary drainage with EUS‐BD for biliary obstructions caused by unresectable pancreatic adenocarcinoma. Moreover, we considered the feasibility of using EUS‐BD as a primary biliary drainage procedure instead of ERCP‐BD.

## Methods

### Patients

**Figure 1:**
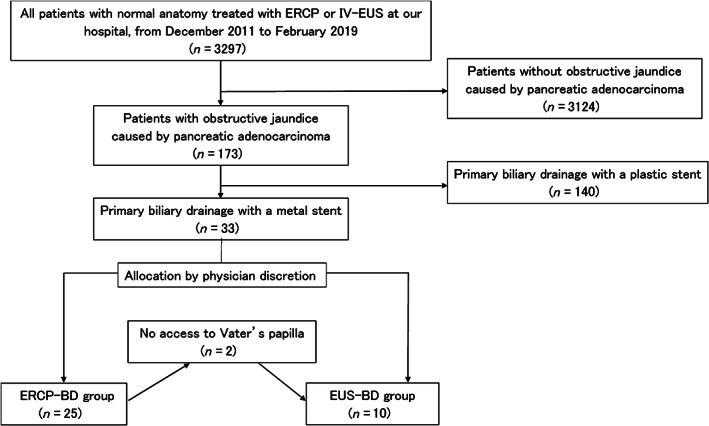
Flow diagram of the patient selection process. ERCP, endoscopic retrograde cholangiopancreatography; ERCP‐BD, endoscopic retrograde cholangiopancreatography‐guided biliary drainage; EUS‐BD, endoscopic ultrasound guided biliary drainage; IV‐EUS, interventional‐endoscopic ultrasound.

Between December 2011 and February 2019, 173 patients aged ≥20 years underwent endoscopic biliary drainage for a biliary obstruction caused by pancreatic adenocarcinoma, in Kawasaki University General Medical Center. For the present study, we enrolled 33 patients who underwent endoscopic biliary drainage with a metal stent in a first attempt of treating obstructive jaundice caused by unresectable pancreatic adenocarcinoma. The patient selection process is illustrated in Figure 1.

Pancreatic adenocarcinoma was detected with computed tomography, magnetic resonance cholangiopancreatgraphy, abdominal ultrasound, and/or EUS. The diagnosis was based on either bile cytology or EUS‐guided fine needle aspiration biopsy. Patients were classified into two groups: those who received ERCP‐BD and those who received EUS‐BD. The outcomes were compared between these two groups.

This study protocol conformed to the 1975 Helsinki Declaration and was approved by the Institutional Research Ethics Committee (Admission No: 3099).

### Procedure

**Figure 2:**
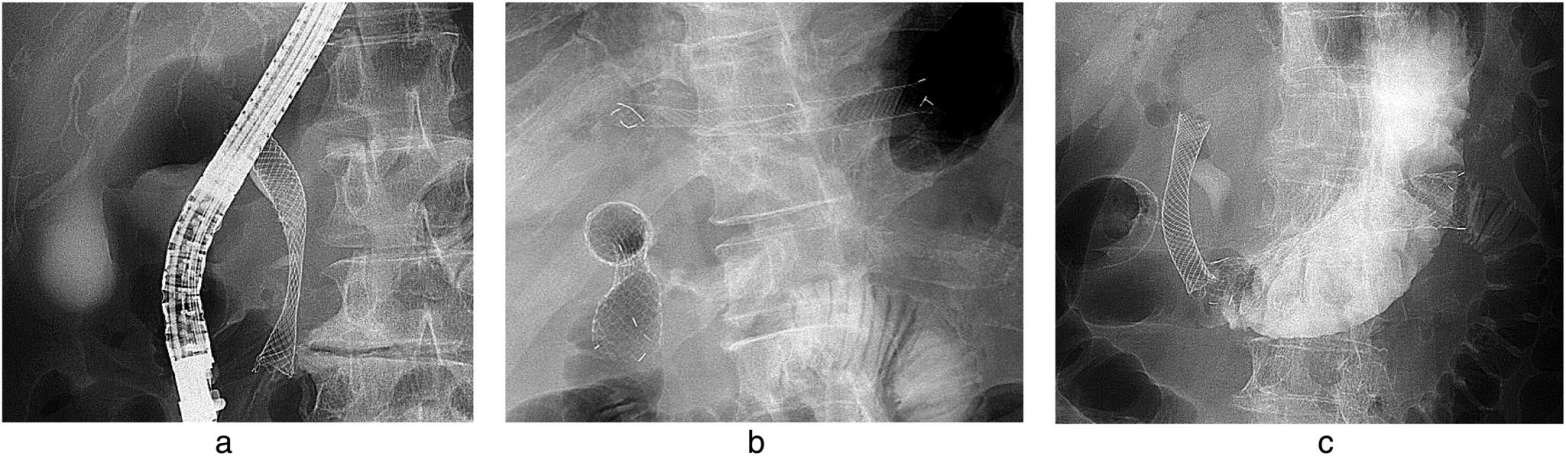
X‐ray images showing different approaches for endoscopic biliary drainage with a metal stent: (a) Endoscopic retrograde cholangiopancreatography‐guided biliary drainage; (b) endoscopic ultrasound‐guided hepaticogastrostomy and a duodenal stent; (c) endoscopic ultrasound‐guided choledocoduodenostomy and a duodenal stent.

We performed ERCP‐BD as the first choice when papillary access was feasible. To perform ERCP‐BD, we used a side‐viewing endoscope (JF‐260V or TJF‐260v; Olympus Co. Ltd., Tokyo, Japan). All patients underwent endoscopic sphincterotomy or endoscopic papillary balloon dilation before stent placement. We selected covered metal stents with diameter >10 mm. The length of the covered metal stent was selected according to the length of the biliary stricture. We inserted the introducer and deployed the covered metal stent across the sphincter of Oddi (Fig. 2a).

When the duodenoscope insertion into the papilla failed, because of gastric or duodenal stenosis or when the covered metal stent could not be completely deployed, we switched to EUS‐BD. In those cases, we typically chose to perform an EUS‐guided hepaticogastrostomy (EUS‐HGS) because most patients had gastric or duodenal stenosis that required a duodenal stent placement. Matsumoto *et al*. reported that biliary stent dysfunction was related to the location of the biliary stent end in double‐stenting cases.^15^ Therefore, ERCP‐BD or EUS‐HGS should be selected for patients with type I or II duodenal stenosis, to avoid interference between the biliary and duodenal stents. However, in the difficult cases, where the intrahepatic duct was punctured under endosonography, we selected EUS‐choledocoduodenostomy (EUS‐CDS). In type III duodenal stenosis with a biliary stricture, EUS‐HGS should also be employed as the initial treatment. In the difficult cases for EUS‐HGS, we selected ERCP‐BD or EUS‐CDS.

We performed the EUS‐HGS with a therapeutic, curved linear‐array echoendocope (GF‐UCT260; Olympus). Briefly, we punctured the intrahepatic bile duct or extrahepatic bile duct with a 19‐G fine needle. After inserting a 0.025‐in. guidewire, we dilated the needle tract with a 6‐mm‐diameter dilation balloon or a diathermic sheath. Finally, we placed a metal stent, with care to prevent stent migration. We used a partially covered metal stent for EUS‐HGS (10 mm in diameter, 10 cm in length) (Fig. 2b) and a fully covered metal stent for EUS‐CDS (10 mm in diameter, 6 cm in length) (Fig. 2c). Additionally, when patients had symptoms related to gastric or duodenal stenosis, the duodenal stent was immediately placed.

### Study outcomes and definitions

The primary outcomes of this study were the technical and clinical success rates of primary biliary drainage. Technical success was defined as a successful metal stent placement after accessing the biliary tract. Clinical success was defined as a >50% reduction in the pretreatment bilirubin value, or an improvement to the normal serum level of bilirubin, with an improvement in acute cholangitis.

The secondary outcomes were stent patency (i.e. the time to recurrent biliary obstruction [RBO]), re‐intervention, and adverse events. The time to RBO was defined as the interval from the time of stent placement to the time of stent occlusion or patient death. Re‐interventions were performed when obstructive jaundice or acute cholangitis occurred as a result of stent dysfunction or dislocation. Adverse events were defined according to the classification of endoscopic adverse events established by the American Society of Gastrointestinal Endoscopy.

### Statistical analysis

All statistical analyses were performed with IBM SPSS Statistics version 26 (IBM, Armonk, NY, USA). We compared continuous variables between groups with the Mann–Whitney *U*‐test, and values are expressed as the median and interquartile range (IQR). Categorical variables were compared with Fisher's exact test. For all analyses, *P* < 0.05 was considered statistically significant.

## Results

### Patient characteristics

This study enrolled 33 patients who underwent endoscopic biliary drainage with a metal stent to relieve biliary obstruction due to unresectable pancreatic adenocarcinoma. Although we planned ERCP‐BDs for 25 patients, in two cases we could not access the major papilla, and therefore we employed EUS‐BDs. Finally, 23 patients underwent ERCP‐BDs, and 10 patients underwent EUS‐BDs. In the EUS‐BD group, EUS‐HGS was performed in nine patients and EUS‐CDS was performed in one patient. Table 1 shows the patient characteristics. At the time of the diagnosis of obstructive jaundice, duodenal stenosis had already occurred in 18 patients. Duodenal stenosis was observed significantly less often in the ERCP‐BD group (*n* = 9/23 patients, 39%) than in the EUS‐BD group (*n* = 9/10 patients, 90%; *P* < 0.01). We found no significant difference between groups in the other characteristics.

**Table 1:** Baseline characteristics of patients with obstructive jaundice caused by unresectable pancreatic adenocarcinoma

	ERCP‐BD group (*n* = 23)	EUS‐BD group (*n* = 10)	*P*‐value
Age (years)	82.0 (70.5–88.5)	76.5 (69.0–84.5)	0.41
Sex: male	8 (35%)	3 (30%)	1.00
EUS‐BD procedure			
EUS‐CDS	—	1 (10%)	
EUS‐HGS	—	9 (90%)	
Duodenal stenosis	9 (39%)	9 (90%)	<0.01
Type of stenosis			
I	8 (35%)	3 (30%)	
II	1 (4%)	5 (50%)	
III	0 (0%)	1 (10%)	
Duodenal stent	5 (22%)	4 (40%)	0.25
T‐Bil (mg/dL)	5.2 (2.7–8.0)	6.9 (4.1–11.4)	0.34
Cholangitis	10 (43%)	2 (20%)	0.26
Pancreatitis	2 (9%)	0 (0%)	1.00
Follow‐up period (days)	87 (24–136)	108 (78–200)	0.33
Prior cholecystectomy	1 (4%)	1 (10%)	0.52
Chemotherapy	7 (30%)	4 (40%)	0.70

Data are presented as the median (interquartile range) or *n* (%).

ERCP‐BD, endoscopic retrograde cholangiopancreatography‐guided biliary drainage; EUS‐BD, endoscopic ultrasound‐guided biliary drainage; EUS‐CDS, endoscopic ultrasound‐guided choledocoduodenostomy; EUS‐HGS, endoscopic ultrasound‐guided hepaticogastrostomy; T‐Bil, total bilirubin.

### Outcomes

The technical success rate was 100% in both groups. The clinical success rate was similar in the ERCP‐BD and EUS‐BD groups (*n* = 21/23 patients, 91% *vs n* = 10/10 patients, 100%; *P* = 0.48). Although the procedure time (from scope insertion to the end of procedure) tended to be shorter in the EUS‐BD group (22 min) than in the ERCP‐BD group (30 min), the difference was not significant (Table 2).

**Table 2:** Outcomes for patients that underwent a primary biliary drainage with a metal stent

	ERCP‐BD group (*n* = 23)	EUS‐BD group (*n* = 10)	*P*‐value
Technical success	23 (100%)	10 (100%)	1.00
Clinical success	21 (91%)	10 (100%)	0.48
Procedure time (min)	30 (19–40)	22 (16–28)	0.18

Data are presented as median (interquartile range) or *n* (%).

ERCP‐BD, endoscopic retrograde cholangiopancreatography‐guided biliary drainage; EUS‐BD, endoscopic ultrasound guided biliary drainage.

RBOs occurred in 6/23 patients (26%) in the ERCP‐BD group and 1/10 patients (10%) in the EUS‐BD group, but the difference between groups was not significant. Symptoms due to RBOs ameliorated after a re‐intervention in all patients.

Adverse events, other than RBOs, were observed in 4/23 patients (17%) in the ERCP‐BD group and in 1/10 patients (10%) in the EUS‐BD group (*P* = 1.00). In the ERCP‐BD group, two patients exhibited bleeding, caused by endoscopic sphincterotomy, and two patients developed acute cholecystitis (Table 3). The bleeding was treated with endoscopic hemostasis. Acute cholecystitis was treated by endoscopically removing the biliary metal stent in one case and by performing EUS‐guided gallbladder drainage in the other case. We did not observe any post‐ERCP pancreatitis. In one patient of the EUS‐BD group, a pseudoaneurysm rupture occurred beside the metal stent end on the intrahepatic bile duct side and was treated with transcatheter arterial embolization.

**Table 3:** Adverse events among patients that underwent a primary biliary drainage with a metal stent

	ERCP‐BD group (*n* = 23)	EUS‐BD group (*n* = 10)	*P*‐value
RBO	6 (26%)	1 (10%)	0.40
Adverse event	4 (17%)	1 (10%)	1.00
Bleeding	2 (9%)	0 (0%)	0.48
Actue cholecystitis	2 (9%)	0 (0%)	0.48
Pseudoaneurysm	0 (0%)	1 (10%)	0.08
Re‐intervention	8 (35%)	1 (10%)	0.22

Data are presented as *n* (%).

ERCP‐BD, endoscopic retrograde cholangiopancreatography‐guided biliary drainage; EUS‐BD, endoscopic ultrasound guided biliary drainage; RBO, recurrent biliary obstruction.

## Discussion

In this study, we evaluated the efficacy and safety of EUS‐BD compared to ERCP‐BD as the primary drainage for malignant biliary stricture caused by unresectable pancreatic adenocarcinoma. The clinical success rates and the incidences of treatment‐related adverse events were comparable between the EUS‐BD and ERCP‐BD groups. These findings were consistent with previous studies, which also demonstrated that ERCP‐BD and EUS‐BD were not significantly different in terms of clinical outcome.^4^, ^6^, ^8^, ^9^, ^10^, ^11^, ^16^, ^17^ Moreover, both our technical and clinical success rates were comparable to those of previous studies. Therefore, our findings support the notion that EUS‐BD could potentially take the place of ERCP‐BD for primary biliary drainage, particularly for patients with specific morbidities.

Pancreatic cancer treatments can be complicated by duodenal stenosis, which makes it difficult to approach the duodenal papilla. When the duodenoscope cannot be inserted through the duodenal stenosis in ERCP‐BD, a duodenal stent must be placed before performing the ERCP‐BD. However, after the duodenal stent placement, the ERCP‐BD was reported to be significantly less technically (56.0%) and clinically (52.0%) successful than a EUS‐BD (95.2, *P* < 0.01, and 90.5%, *P* = 0.01, respectively).^18^ Moreover, a previous study showed that both the biliary stent for ERCP‐BD and the duodenal stent for EUS‐CDS had significantly shorter patency times than the duodenal stent placed for EUS‐HGS (107 *vs* 270 days, *P* = 0.029).^15^ Accordingly, EUS‐BD, and particularly EUS‐HGS, should be chosen as the primary biliary drainage method for patients with both types of stentosis, whether they occur simultaneously or heterochronously.

In this study, the procedure time was not significantly different between groups, but EUS‐BD tended require a shorter procedure time than ERCP‐BD. One reason for this result could be that trainee endoscopists were included only in the ERCP‐BD group. Therefore, we could not conclude whether EUS‐BD could shorten procedure time. However, some studies have reported that the procedure time for EUS‐CDS was significantly shorter than that required for ERCP‐BD.^7^, ^12^ These results suggested that, in light of technical success, we could choose EUS‐BD as the primary biliary drainage method for patients with concurrent morbidities.

In this study, the incidence of adverse events was not significantly different between the groups. Some previous studies had also shown that the incidence of adverse events was not significantly different between ERCP‐BD and EUS‐BD treatment groups.^4^, ^6^, ^8^, ^9^, ^10^, ^11^, ^16^, ^17^ However, some other previous studies had reported that EUS‐BD induced severe adverse events, but the incidence was rare: only 0–2.1%.^2^, ^7^, ^11^ In our study, the most severe adverse event was major bleeding due to pseudoaneurysm rupture. This event occurred in one patient in the EUS‐BD group. However, in previous studies, this adverse event also occurred when metal stents were used in patients treated with ERCP‐BD or PTBD.^19^, ^20^, ^21^ The mechanism underlying the development of a pseudoaneurysm due to metal stents remains unclear. Previously, Satoh *et al*. suggested that various factors might be associated with the development of a pseudoaneurysm, including mucosal necrosis due to cancer invasion, luminal compression by the stents, high compression on the arterial wall due to the expansion of the metallic stent, or unknown factors.^19^ Prachayakul *et al*. reported that pseudoaneurysm ruptures due to EUS‐HGS were very rare.^20^ In this study, the patient with a pseudoaneurysm associated with EUS‐HGS had symptoms of hematemesis and was rescued by transcatheter arterial embolization. When a patient undergoing EUS‐HGS displays hematemesis, a pseudoaneurysm rupture should be suspected as a potential cause of bleeding.

In our study, post‐ERCP pancreatitis did not occur in the ERCP‐BD group, despite the fact that it is a common adverse event after ERCP‐associated procedures. Some previous studies had shown that, compared to ERCP‐BD, EUS‐BD could reduce the rates of acute pancreatitis and re‐intervention.^6^, ^9^, ^12^, ^17^

In the present study, two patients in the ERCP‐BD group developed cholecystitis, which required metal stent removal and EUS‐guided gallbladder drainage. Previously, Nakai *et al*. reported that tumor involvement at the orifice of the cystic duct was a risk factor for cholecystitis after a metal stent placement.^22^ In this study, both cases of cholecystitis occurred in patients with tumor involvement at the orifice of the cystic duct. This result suggested that EUS‐BD might be preferable to ERCP‐BD in patients with tumor involvement at the cystic duct.

This study had several limitations. The study design was retrospective in nature, we included a small number of cases, and it was conducted at a single center. Another limitation was that the endoscopists were given the choice of performing ERCP‐BD or EUS‐BD as the primary drainage procedure for patients with malignant biliary strictures that were suspected to get complicated with duodenal stenosis in the near future. Furthermore, there were no cases of patients who received ERCP‐BD after EUS‐BD failure. Finally, the EUS‐BD procedures were performed only by expert endoscopists, but the ERCP‐BD procedures were performed by endoscopists at any level of expertise, including trainees.

In conclusion, our findings demonstrated that the EUS‐BD could serve as the primary biliary drainage option for obstructive jaundice caused by unresectable pancreatic adenocarcinoma. This approach could be particularly favorable in patients with duodenal stenosis or tumor involvement at the orifice of the cystic duct. Although the EUS‐BD approach might not completely replace the ERCP‐BD approach, our data and previous reports suggest that the EUS‐BD approach could be applied to more patients with pancreatic adenocarcinoma, without first attempting a ERCP‐BD.
